# Rapid urbanization - Its impact on mental health: A South Asian perspective

**DOI:** 10.4103/0019-5545.43623

**Published:** 2008

**Authors:** Jitendra K. Trivedi, Himanshu Sareen, Mohan Dhyani

**Affiliations:** Department of Psychiatry, CSM Medical University (Earlier KG Medical University), Lucknow - 226 003, India

**Keywords:** Mental health, South Asia, urbanization

## Abstract

Rapid increase in urban population as a proportion of total population is resulting in rapid urbanization of the world. By the end of 2008, a majority of the world's population will be living in the cities. This paradigm shift in the dynamics of human population is attracting attention of demographers, sociologists, scientists, and politicians alike. Urbanization brings with it a unique set of advantages and disadvantages. Though it is driving the economies of most of the nations of the world, a serious concern regarding the impact of urbanization on mental health is warranted. The impact of urbanization on mental health in South-Asian countries needs to be examined. These countries by virtue of their developing economies and a significant proportion of population still living below poverty line are particularly vulnerable and tend to have a higher burden of diseases with an already compromised primary health care delivery system. The range of disorders and deviancies associated with urbanization is enormous and includes psychoses, depression, sociopathy, substance abuse, alcoholism, crime, delinquency, vandalism, family disintegration, and alienation. Thus, it is a heterogenous mix of problems and categorizing them to one particular subtype seems daunting and undesirable. Urbanization is affecting the entire gamut of population especially the vulnerable sections of society - elderly, children and adolescents, and women. Rapid urbanization has also led to creation of “fringe population” mostly living from hand to mouth which further adds to poverty. Poverty and mental health have a complex and multidimensional relationship. Urban population is heavily influenced by changing cultural dynamics leading to particular psychiatric problems like depression, alcoholism, and delinquency. Judicious use of resources, balanced approach to development, and sound government policies are advocated for appropriate growth of advancing economies of South-Asian region.

Urbanization is the relative increase of the urban population as a proportion of the total population[1] and it is occurring on a scale never before experienced. The United Nation's Population Fund (UNFPA) released their “State of the World Population 2007” report in June 2007[2] which mentions the fact that humanity is nearing the date when for the first time more humans will be living in cities than in rural areas. The report mentions this watershed event, which demographers predict will occur sometime in 2008, as the most important trend in human development. The vast majority of these new urban dwellers will live in developing countries (like those in the South-Asian region), and they will be poor. This will present major challenges for the nations least prepared to meet the inevitable strains of urban growth.

Cities offer the lure of better employment, education, health care, and culture; and they contribute disproportionately to national economies.

However, rapid and often unplanned urban growth is often associated with poverty, environmental degradation, and population demands that outstrip service capacity. These conditions place human health at risk. Reliable urban health statistics are largely unavailable throughout the world. Disaggregated intra-urban health data, i.e., for different areas within a city, are even rarer.

Data that are available indicate a range of urban health hazards and associated health risks: substandard housing, crowding, air pollution, insufficient or contaminated drinking water, inadequate sanitation and solid waste disposal services, vector-borne diseases, industrial waste, increased motor vehicle traffic, stress associated with poverty and unemployment, among others. Local and national governments and multilateral organizations are all grappling with the challenges of urbanization.

Urbanization has brought its own set of problems pertaining to mental health and well-being. Mostly because of increased speed and decreased costs of communication and transportation, cities are growing increasingly diverse in their population. Consequently, cultural factors have taken center stage in the understanding of urban mental health.

It is often thought whether the increased scale and proportion of the cities are exceeding human capabilities to live under conditions of security and mutual support and concern. Some feel the sheer scale of urban life is forcing individual identity to yield to anonymity, indifference, and narrow self-interest. Commentaries on the growing fear, powerlessness, and anger of urban residents are numerous.

The multiculturalism of today's cities contributes to increased tolerance, better quality of life, and sociocultural stimulation; at the same time, it often contributes to heightened social tensions, interethnic striving, and cultural conflicts - all of which undoubtedly carry mental health ramifications. The range of disorders and deviancies associated with urbanization is enormous and includes psychoses, depression, sociopathy, substance abuse, alcoholism, crime, delinquency, vandalism, family disintegration, and alienation. Such negative impact often results in unreasonable means which may result in communal violence.[3] Negative impact is also experienced by behavior constraints practiced or imposed upon the urban people. If behavior is unduly suppressive, it may result in learned helplessness leading to stress-related disorders.[4] Conflicts, wars (e.g., in Afghanistan), and civil strife (e.g., in Pakistan and Myanmar currently) in the deprived countries cause higher rates of mental health problems (as reflected in increased rates of post-traumatic stress disorder [PTSD], anxiety, and depressive disorders).

Migration to cities has increased dramatically over the past few decades. Most migrants come from rural areas, bringing values, beliefs, and expectations about mental health that are often very different from the ones they encounter in their new location. In many instances, people coming from rural areas have endured years of isolation, lack of technologic connection, poor health, poverty, unemployment, and inadequate housing. They need to acculturate and adapt not only to a new challenging urban environment, but also to alternative systems of symbols, meanings, and traditions.

There have been suggestions that social deviance could be traced to many of the social processes accompanying urbanization, including competition, class conflict, accommodation, and assimilation.[5]

## SOUTH ASIA: A UNIQUE PERSPECTIVE

South Asia is the most heavily populated and among the poorest regions in the world. It faces enormous social, economic, and health challenges, including pervasive inequality, violence, political instability, and limited resources.

These countries tend to have a higher burden of diseases and have an already compromised primary health care delivery system. They are also plagued by lack of awareness of the constituting population, stigma associated with mental illness, poverty, and illiteracy.

Rapid urbanization puts immense pressure on both physician and patient. Sound doctor-patient relationship becomes almost impossible in busy clinics. In view of large number of patients, time constraints and wide range of diseases, making accurate diagnosis and proper follow-up are almost impracticable. This leads to a high rate of clinical errors and superfluous diagnostic tests.

## DATA FROM SOUTH ASIAN COUNTRIES

1. In India, as per the 2001 census, 72.22% of the people live in more than 550,000 villages, and the remainder in more than 2000 towns and cities.
2. In Pakistan, about 68% of the population lived in rural areas in 1994, a decrease of 7% since 1970. In contrast, the number of people living in urban areas has risen substantially, resulting in an urban growth rate of 4.6% between 1980 and 1991.
3. The population of Nepal is overwhelmingly rural, with just over 15% living in urban areas such as the Kathmandu Valley.
4. The distribution of rural population in other South Asian countries is as follows:

Bhutan 91%, Sri Lanka 79%, Afghanistan 77%, Maldives 71%, and Myanmar 71% (source www.nationmaster.com).

Mental health resources both in manpower and facilities are extremely scarce and maldistributed in developing countries in Asia, e.g., 35,000 psychiatrists for 3 billion people (USA 55,000 psychiatrists for 285 million).

## OLD AGE AND URBANIZATION: CHANGING DYNAMICS

Urbanization alters the dynamics of society at large and family in particular. Rapid urbanization has created a huge population of older men and women left to fend for themselves in the rural areas while the young make their living in the cities. This also means less availability of caregivers when older people fall ill.

By 1990, a clear majority (58%) of the world's population aged 60 years and over was already to be found living in developing countries. By 2020, this proportion will have risen to 67%. Over this period of 30 years, this oldest sector of the population will have increased in number by 200% in developing countries as compared to 68% in the developed world. This demographic transition will be accompanied by unprecedented economic growth and industrialization, and by profound changes in social organization and in the pattern of family life.

Among the neuropsychiatric conditions, dementia and major depression are the two leading contributors, accounting, respectively, for one-quarter and one-sixth of all disability adjusted life years (DALYs) in this group. Most people with dementia live in developing countries: 60% in 2001, rising to 71% by 2040. Rates of increase are not uniform: numbers are forecast to increase by 100% in developed countries between 2001 and 2040, but by more than 300% in India, China, and their South Asian and Western Pacific neighbors.

Developing country health services are generally ill-equipped to meet the needs of older persons. Health care, even at the primary care level, is clinic-based: the older person must attend the clinic, often involving a long journey and waiting time in the clinic, to receive care. Even if they can get to the clinic, the assessment and treatment that they receive is orientated toward acute rather than chronic conditions. The perception is that the former may be treatable, the latter intractable and not within the realm of responsibility of health services.

In developed countries, with their comprehensive health and social care systems, the vital caring role of families, and their need for support, is often overlooked. Conversely, in developing countries, the reliability and universality of the family care system is often overestimated; older people are among the most vulnerable groups, in part because of the continuing myths that surround their place in society.

## CHILDREN, MENTAL HEALTH, AND URBANIZATION

Children and women are especially vulnerable to interpersonal violence in urban areas, especially in developing countries, where cities are populated by a large percentage of children and adolescents. By 2025, 6 of 10 children will live in cities. As a result of rural-urban migration and high fertility rates, it is estimated that about 50% of the urban population in developing countries is younger than 25 years. In addition, there are approximately 30 million street children worldwide, and most of them are involved in illegal activities in urban areas.

Children and adolescents in socioeconomically deprived urban areas are often drawn to antisocial behavior. Although not exclusively an urban phenomenon, it thrives in inner cities where degradation, poverty, drug use, and unemployment result in an explosive blend favoring violent solutions.

## WOMEN AND MENTAL HEALTH: IMPACT OF URBANIZATION

Women are particularly vulnerable and they often disproportionately bear the burden of changes associated with urbanization. In the rural setup, they would work mostly at homes but the predominantly nuclear setup of the cities and sheer economics is forcing women to venture out. Domestic violence is also highly prevalent in urban areas. In both developed and developing countries, women living in urban settings are at greatest risk to be assaulted by intimates.[6] A meta-analysis of 13 epidemiological studies in different regions of India revealed an overall prevalence rate of mental disorders in women of 64.8 per 1000.[7] Women had significantly higher prevalence rates for neuroses, affective disorders, and organic psychoses than men. A survey carried out in Nepal demonstrated that women had a higher psychiatric morbidity than men, with a sex ratio of 2.8:1 in the health post, and 1.1:1 in the district hospital.[8] A study in Bangladesh showed that the sex ratio for mental disorders was 2:1 and that for suicide was 3:1.[9]

In deprived countries, women bear the burden of responsibilities of being wives, mothers, educator, and carers; at the same time a part of labor force. In 25-33% households, they are the prime source of income. Significant gender discrimination, malnutrition, overwork, domestic, and sexual violence add up to the problems. Social support and the presence of close relationships (more commonly observed in rural society) appear to be protective against violence. The women although have a greater role to play in the urban setup, but the rise in hierarchy in society that should rightfully accompany this increased demand on them is still missing.

The rate of mental distress has been reported to be high also in working women in South-East Asian countries and cultural factors are among the contributing variables.[10] This mental distress usually remains unacknowledged.[11]

### Urbanization and poverty

Rapid urbanization has led to creation of “fringe population” mostly living from hand to mouth which further adds to poverty. Poverty and mental health have a complex and multidimensional relationship. Poverty is understood as lack of both social and educational resources. Poor and the deprived nations (like most nations in South-Asian region) have a high prevalence of mental and behavioral disorders by either the social causation theory or the social drift theory. In the absence organized social welfare agencies, in the deprived countries, vicious cycle and impoverishment progress.

**Figure 1:**
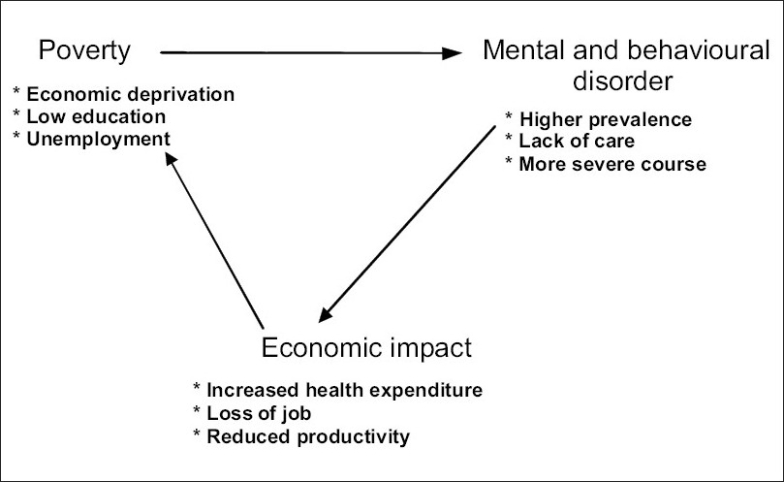
Inter-relation of economic status and mental and behavioral disorders

Low socioeconomic status is known to be associated with a higher prevalence of major depression, substance abuse, and personality disorders [Figure 1].

### Urbanization and psychiatry

Using a composite diagnostic interview, World Health Organization (WHO) investigators studied cross-national comparisons of the prevalence and correlates of mental disorders. They found a consistent pattern of higher prevalence of mental disorders in urban areas than in rural areas.

Cultural determinants, such as attitude toward persons with mental illness, play a major role in the drifting of untreated individuals toward the lower layers of society, which may significantly hamper chances of reintegration. The relationship with social rank is also an important determinant of physical and mental health and is heavily influenced by cultural dynamics.

The increased opportunities for geographic mobility have produced an unprecedented multiethnic influx to cities. The complexities of cultural aspects that impact psychopathology and mental health are producing both challenging and beneficial changes in the way psychiatry is practiced. The negative aspects of such multiethnic migration, however, may include lack of familiarity with illness presentation, culturally specific belief systems, and reluctance to rely on medical systems - all of which may significantly delay proper assessment and treatment.

Research on the relationship between urban living and schizophrenia has yielded culturally intriguing findings. The international pilot study for schizophrenia compared 1200 patients in nine countries.[12] The investigators found that patients with schizophrenia in developing countries tended to have a less severe course and better outcomes than those in developed countries and those outcomes may be more favorable in rural settings. Favorable outcome was associated with vertical mobility, extended families, psychiatric services that included active family participation, and absence of specific community stereotypes of mentally ill persons. These findings point to the importance of cultural expectations, support systems, and stigma. High tolerance for mental illness appears to have a significantly positive impact on patients with schizophrenia in developing countries.

Similarly, in the outcome of severe mental disorders study in patients with schizophrenia,[13] all measured indices had better outcomes in developing countries than in developed ones. A particularly striking finding was that 41.6% of the sample from the developed-countries cohort had impaired social functioning throughout the follow-up period, compared with 15.7% of the sample from developing countries. How much of this large difference can be accounted for by the local cultural expectations for functioning remains an unanswered question that awaits further inquiry.

Chronic difficulties such as poor, overcrowded physical environments, high levels of violence and accidents, insecure tenure, and poor housing have all been shown to be associated with depression. In developing countries, major depression is projected to be the leading cause of disease burden.[14]

## CONCLUSIONS

In an editorial on urban health issues in The Lancet, it was stated that “…to keep cities profitable into the 21st century more attention will need to be paid to aspects of health other than the purely physical.”[15] Mental ill-health is a growing problem and one that urgently requires attention.

For the problem of mental ill-health to be successfully confronted, a broad understanding of poverty, taking into account both individual and contextual factors, is required. Such an approach is in line with current thinking in urban health research which suggests that an integrated approach, and one that acknowledges the complexity of urban health problems, is the way forward.[16]

The government of these countries should be encouraged to adopt sound mental health policies, to allocate budget according to comprehensive assessment of the morbidity and cost of the illnesses to society, policies should be according to the needs of different populations and the use of locally available resources should be encouraged and utilized to the hilt.

Urbanization is not all bad or all evil. It may be prudent to say that urbanization is driving the economies of all the countries in the South-Asian region and enabling them to surge ahead from strength to strength. The UNFPA report mentions “no country in the industrial age has ever achieved significant economic growth without urbanization.” Need of the hour is judicious use of resources, balanced approach to development, and sound government policies.
